# Analysis of cuproptosis in hepatocellular carcinoma using multi-omics reveals a comprehensive HCC landscape and the immune patterns of cuproptosis

**DOI:** 10.3389/fonc.2022.1009036

**Published:** 2022-11-02

**Authors:** Xinqiang Li, Peng Jiang, Ruixia Li, Bin Wu, Kai Zhao, Shipeng Li, Jinzhen Cai

**Affiliations:** ^1^ Organ Transplantation Center, Affiliated Hospital of Qingdao University, Qingdao, China; ^2^ Institute of Organ Donation and Transplantation, Medical College of Qingdao University, Qingdao, China; ^3^ Department of Pulmonary and Critical Care Medicine, The First Hospital of China Medical University, Shenyang, China; ^4^ The Second Clinical Medical College, Capital Medical University, Beijing, China

**Keywords:** cuproptosis, cell death, omics, HCC, single cell RNA analysis

## Abstract

Cuproptosis represents a novel copper-dependent regulated cell death, distinct from other known cell death processes. In this report, a comprehensive analysis of cuproptosis in hepatocellular carcinoma (HCC) was conducted using multi-omics including genomics, bulk RNA-seq, single cell RNA-seq and proteomics. ATP7A, PDHA1 and DLST comprised the top 3 mutation genes in The Cancer Genome Atlas (TCGA)-LIHC; 9 cuproptosis-related genes showed significant, independent prognostic values. Cuproptosis-related hepatocytes were identified and their function were evaluated in single cell assays. Based on cuproptosis-related gene expressions, two immune patterns were found, with the cuproptosis-C1 subtype identified as a cytotoxic immune pattern, while the cuproptosis-C2 subtype was identified as a regulatory immune pattern. Cuproptosis-C2 was associated with a number of pathways involving tumorigenesis. A prognosis model based on differentially expressed genes (DEGs) of cuproptosis patterns was constructed and validated. We established a cuproptosis index (CPI) and further performed an analysis of its clinical relevance. High CPI values were associated with increased levels of alpha-fetoprotein (AFP) and advanced tumor stages. Taken together, this comprehensive analysis provides important, new insights into cuproptosis mechanisms associated with human HCC.

## Introduction

Hepatocellular carcinoma (HCC) represents the most common type of primary liver cancer (1), ranking as the fourth leading cause of tumor-related deaths worldwide (2). Most HCC patients show poor outcomes due to the difficulties of early diagnosis and treatment of advanced HCC (3). Additional factors contributing to low survival rates as associated with HCC include high probabilities for recurrence and metastasis after surgical treatment (4), high tumor heterogeneity of HCC resulting in drug resistance and limited efficacy of systemic therapies (5). Therefore, identifying novel and reliable methods to enhance the efficacy of diagnosis and treatment is urgently needed to improve long-term outcomes of HCC.

While there exist numerous studies on the mechanisms involved with cell death from HCC, a principal conclusion resulting from these reports is that these mechanisms are complex. With HCC, as well as in other tumors, various types of cell death are associated with the biological behavior of these tumor cells (6, 7). Cuproptosis represents a novel, copper-dependent regulated cell death (8, 9), which is distinct from other known cell death processes such as apoptosis (10), necroptosis (11), pyroptosis (12) and ferroptosis (13). Cuproptosis was recently found to involve a direct binding of copper to lipoylated components of the tricarboxylic acid (TCA) cycle with the subsequent aggregation and loss of lipoylated proteins leading to cell death (14). A number of genes associated with cuproptosis have been identified including FDX1, LIPT1, LIAS, DLD, DLAT, PDHA1, PDHB, DBT, GCSH, DLST, SLC31A1, ATP7A and ATP7B. In particular, FDX1, which encodes a reductase to transfer Cu^2+^ to Cu^1+^, is thought to be a key regulator of cuproptosis and an upstream regulator of protein lipoylation (8, 15).

The issue as to whether cuproptosis related genes influence tumor microenvironments and the means through which it may impact the prognosis of HCC remains unknown (16). Given the severity of HCC, an urgent need exists to achieve a comprehensive understanding of the relationship between cuproptosis and HCC. Such information would be critical in elucidating the immune patterns and identifying potential treatment targets for HCC. In the present study, detailed analysis of cuproptosis related genes was performed, as achieved using genomics, bulk RNA-seq and single cell RNA-seq to dissect a cluster of cuproptosis related hepatocytes in HCC. Then, two immune clusters with distinct gene patterns based on cuproptosis-related genes were constructed. Lastly, we identified a prognostic gene signature in the TCGA cohort and validated this gene signature in the International Cancer Genome Consortium (ICGC) cohort.

## Methods

### HCC datasets and preprocessing

The workflow of this study is summarized in **Figure S1A**. For bulk RNA-seq, gene expression data and clinical metadata from HCC samples were downloaded from the GDC TCGA Liver Cancer (LIHC, n=421) (https://xenabrowser.net/datapages/) and ICGC Data Portal (https://dcc.icgc.org/releases/current/Projects/) named LICA-FR Liver Cancer - FR (N=161). These sites provide data on gene expression, clinical information and survival phenotype, which were then used for further analysis. For single cell RNA-seq, we downloaded data from the Gene Expression Omnibus (GEO) repository (https://www.ncbi.nlm.nih.gov/geo/) and the accession ID was GSE156625 (17), which contains 14 pairs of tumor samples of human HCC with a 10X genomics platform (**Table S1**).

### Gene mutation analysis

Gene mutation data, including somatic mutations and copy number variation (CNV) in HCC were downloaded from the GDC TCGA Liver Cancer (https://xenabrowser.net/datapages/) and cBioPortal for Cancer Genomics (http://www.cbioportal.org/) sites. The maftools R package (version 2.6.05) (18) was used to perform the analysis and visualize somatic variants of HCC. In addition, the RCircos R package (version 1.2.2) (19) was utilized to plot the CNV atlas of cuproptosis-related genes in human chromosomes.

### Pathway enrichment analysis

Metascape (20), a webtool for gene annotation and analysis, was employed to perform pathway enrichment analysis and protein-protein interaction (PPI). We also employed the functional enrichment analysis using the clusterProfiler package (version 3.17.0) (21) and org.Hs.eg.db package (version 3.11.4) for Gene Ontology (GO) and Kyoto Encyclopedia of Genes and Genomes (KEGG) analysis to highlight biological processes and potential functions of genes. Both GO and KEGG used a p < 0.05 as their probability limit value. Results were visualized using barplot or dotplot functions.

### Correlation analysis

The ggcorrplot package (version 0.1.3) was used to calculate and visualize potential correlations among cuproptosis-related genes according to gene expressions present in HCC.

### Study subjects

A total of 20 pairs of tumor and adjacent tissue from HCC patients were collected from the Affiliated Hospital of Qingdao University. This study was approved by the ethics committee of Affiliated Hospital of Qingdao University.

### Immunohistochemical staining analysis

Paraffin sections are routinely dewaxed to hydration, and washed with distilled water. Following incubation in 3%H2O2 for 10min, antibodies anti-DBT, DLD, FDX1 and SCL31A1 were added and incubated at 4°C for overnight. The specimens were incubated with secondary antibodies at 37°C for 1 h, followed by diaminobenzidine staining.

### Quantitative PCR

TRIzol reagent (Invitrogen, USA) was used to extract the total RNAs of tissues. RNA samples were reversely transcribed into cDNA by using the ABScript III RT Master Mix for qPCR with gDNA Remover (ABclonal). All results were processed with GAPDH for standardization. Relative quantification analysis was performed using the comparative CT (2−ΔΔCT) method.

### Western blot

Tissue specimens were ground with a tissue grinder and lysed using RIPA lysis buffer on ice for 1 h.Proteins were electrophoresed on 12% SDS-PAGE gels and blocked with 5% nonfat dry milk in TBST configuration for 1 h at room temperature after membrane transfer.Antibodies used were DLD (Abclonal, A5220), DBT(Abclonal,A20381).Final membranes were developed with ECL luminescent liquid.

### Survival analysis

Survival analysis was conducted as based on the survival data of TCGA LIHC using the survival (version 3.2.3) and survminer (version 0.4.8) R package with default parameters, regarding levels of gene expression.

### Preprocessing of scRNA-seq data

Single cell analysis was carried out following the previous study (22). The Seurat R package (version 3.2.0) (23), a tool for single cell genomics, was utilized to process single cell RNA sequencing data. Cells with < 5% mitochondrial counts were filtered, with a total of 73,589 high quality cells then available for use in downstream analysis. Cells were normalized and scaled with the default parameters. Highly variable features were identified using FindVariableFeatures function and we then performed principal components analysis (PCA) analysis with the determined variable features. Dimension reduction and clustering were conducted using FindNeighbors (dims = 1:10) and FindClusters (resolution = 0.5) functions. A non-linear dimensional reduction (UMAP) was then run to assess and visualize the data.

### Differentially expressed genes as identified from scRNA-seq data

We found 29 clusters in the single cell landscape of HCC. To identify differentially expressed features according to clusters, cell types and tissue locations, we performed analyses using FindMarkers and FindAllMarkers functions. The threshold of logFC was 0.25 while the minimum fraction of genes detected in cells was 0.1, with default.

### Cell type annotation

Canonical marker genes (**Table S2**) were used to annotate cell types of the 29 clusters. For the entire atlas, cells were annotated as Hepatocytes, Endothelial cells, Fibroblasts, CD4+ T cells, CD8+ T cells, regulatory T (Treg) cells, B cells, Myeloid cells, natural killer (NK) cells, Mast cells and Bi-potent cells. We also combined the original annotation results and used the SingleR package (version 1.2.4) (24) to help identify the cell types.

### Pseudotime analysis

To identify the potential evolution process of hepatocytes, we performed a trajectory analysis using the monocle package (version 2.17.0) (25) with the following parameters: lowerDetectionLimit=0.5, min_expr=0.1 and num_cells_expressed>= 10. Results were visualized with use of a plot_cell_trajectory function according to pseudotime and seurat clusters.

### Consensus molecular clustering and PCA

We established the existence of 13 cuproptosis-related genes within our latest study (8) and performed consensus clustering using the ConsensusClusterPlus package (version 1.54.0) (26) as based on the expression of these 13 cuproptosis-related genes in GDC TCGA Liver Cancer (LIHC, N=421). After a comprehensive assessment of these results, including consensus matrices, consensus cumulative distribution function (CDF) plot and item-consensus plot, two clusters were finally identified. In addition, we performed PCA to visualize the discrepancy between the two clusters using the ggord function.

### Gene set variation analysis

Gene set variation analysis (GSVA) provides an indication for use of a particular technique for gene set enrichment. Accordingly, the GSVA package (version 1.38.2) (27) and GSVAdata package (version 1.26.0) were employed to evaluate potential differences in pathway activity between the two different patterns. The reference gene set was downloaded from the Molecular Signatures Database (MSigDB) (version 7.4).

### Estimation of immune infiltration

To estimate the immune infiltration of samples, we used a single sample gene set enrichment analysis (ssGSEA). With this analysis it is possible to profile immune cell infiltration patterns and evaluate specific cell types between the two clusters. Another deconvolution approach, CIBERSORT (http://cibersort.stanford.edu/) (28), was applied to estimate the abundance of 22 distinct cell subsets according to the gene expression. We also used the estimate package (version 1.0.13) to infer immune and stromal cell admixture for bulk RNA-seq data.

### TIDE and immune checkpoint analysis

Tumor immune dysfunction and exclusion (TIDE) score was first developed by Jiang et al (29), which has been proven to have remarkable power for predicting the prognosis of cancer patients. We acquired TIDE score, merck18 (T-cell-inflamed signature) score, CD8 score, dysfunction score, and exclusion score from the TIDE web (http://tide.dfci.harvard.edu).

### Establishment and validation of the prognostic model

DEGs between two immune clusters of TCGA-LIHC samples were identified using the limma package (version 3.45.9) with a cut off criteria of adj.p.value < 0.05 and |logFC| > 1.5. Univariate Cox regression was carried out to evaluate the prognostic effect of DEGs in the TCGA-LIHC cohort with a passing criteria requiring a p value < 0.05 and Hazard ratio > 1. A stepwise multiple regression analysis was then applied for the DEGs using the survival (version 3.2.3) and survminer (version 0.4.8) packages. The ICGC-LIHC datasets were used to validate the power of the prognostic model. The risk score was calculated in the training and testing sets with use of the following formula:


$$\text{Survival risk score }\left(\text{SRS}\right)\;=\sum_{i=1}^{i=n}C_{i}\cdot \nu_{i}$$

Where n indicates the number of mRNAs in the prognostic model, ci the coefficient of the mRNA included and vi their expression level.

The high-risk groups and low-risk groups in various cohorts were divided by the optimal cutoff point which was calculated by the “surv_cutpoint” function in the “survminer” package (version 0.4.8).

### Construction of cuproptosis scores

A cuproptosis scoring scheme was developed to evaluate cuproptosis levels within each patient using PCA. DEGs identified from two immune clusters of TCGA-LIHC samples were used to perform the univariate Cox regression. Genes with significant survival value were then chosen for further selection using the rfe function with random forest and the 10-fold cross validation technique in the caret R package (version 6.0-91). Next, PCA was performed as based on the expression level of selected genes and principal components 1 and 2 were extracted to serve as the signature score. Similar to previous studies (30, 31), we defined the cuproptosis score as = Σ(PC1i+PC2i).

### Drug sensitivity prediction

Based on the Genomics of Drug Sensitivity in Cancer (GDSC) database (32), we carried out drug sensitivity prediction using the pRRophetic package (version 0.5), where the half-maximum inhibitory concentration (IC50) of each patient was estimated using Ridge’s regression. The accuracy of the prediction was estimated by 10-fold cross validation.

### Statistical analysis

Student t-tests were utilized to compare gene expression levels between tumor and normal tissue samples. Mann-Whitney U-tests with P values adjusted by the BH method were used to compare the ssGSEA score of immune cells or pathways between groups. A Kaplan-Meier analysis with the log-rank test was performed to evaluate the OS of each group. Univariate Cox regression analyses were employed to assess the hazard ratio (HR) of cuproptosis subtype-related genes. Multivariable cox regression analysis was conducted to determine the independent prognostic factors and construct the prognostic signature. The efficacy of the prognostic signature was evaluated by the area under the curve (AUC) calculated by the R package “pROC”. To compare the gene expression of pan-cancer, we used the TIMER2.0 tool (33) to calculate and visualize the TCGA database. All statistical analyses were performed using R software (version 4.0.2) and its appropriate packages. P values <0.05 were considered as statistically significant.

## Results

### Landscape of genetic and transcriptional alterations of cuproptosis-related genes in HCC

**Figure 1:**
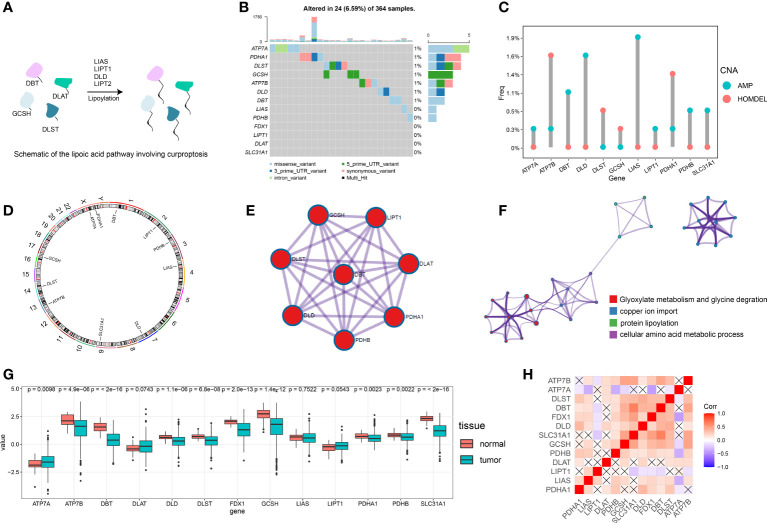
Overview of the multi-omics analysis for cuproptosis. **(A)** Schematic diagram for the lipoic acid pathway involving cuproptosis. **(B)** Of the 364 patients with HCC, 24 (6.59%) showed gene mutations in 13 cuproptosis-related genes, primarily including missense variants, 5’ UTR variants and synonymous variants. **(C)** The CNV mutation was prevalent in cuproptosis-related genes. Columns represent the alteration frequency, blue dots the amplification frequency and red dots the deletion frequency. **(D)** Location of the CNV alteration in cuproptosis-related genes on the chromosome. **(E)** Protein-protein interactions of cuproptosis-related genes. **(F)** Metascape network visualization showing enrichment pathway terms. Cluster annotations are color coded. **(G)** Differences in gene expression levels for each cuproptosis-related gene between normal and tumor tissues. **(H)** Visualization of the gene correlation matrix. “X” represents a lack of statistical significance.

The roles of 13 cuproptosis-related genes (FDX1, LIPT1, LIAS, DLD, DLAT, PDHA1, PDHB, DBT, GCSH, DLST, SLC31A1, ATP7A, ATP7B) in HCC were investigated (**Table S3**). **Figure 1A** contains a summary of additional genes, other than FDX1, that are essential to the lipoic acid pathway and critical mediators of copper ionophore–induced cell death.

We first assessed the prevalence of somatic mutations of these 13 cuproptosis-related genes in HCC. The overall mutation of all cuproptosis-related genes is relatively low in the HCC genome. A total of 24 of 364 samples (6.59%) demonstrated genetic alterations in cuproptosis-related genes, primarily consisting of missense variants, 5’ UTR variants and synonymous variants (**Figure 1B**). ATP7A showed the greatest amount of variant frequency, followed by PDHA1 and DLST. Moreover, analysis of these 13 cuproptosis-related genes revealed that CNV alterations were prevalent. DBT, DLD, LIAS, PDHB and SLC31A1 showed widespread CNV amplification while ATP7B, DLST, GCSH and PDHA1 showed prevalent CNV deletions (**Figure 1C**). Locations of CNV alterations in cuproptosis-related genes in HCC are presented in **Figure 1D**.

**Figure 2:**
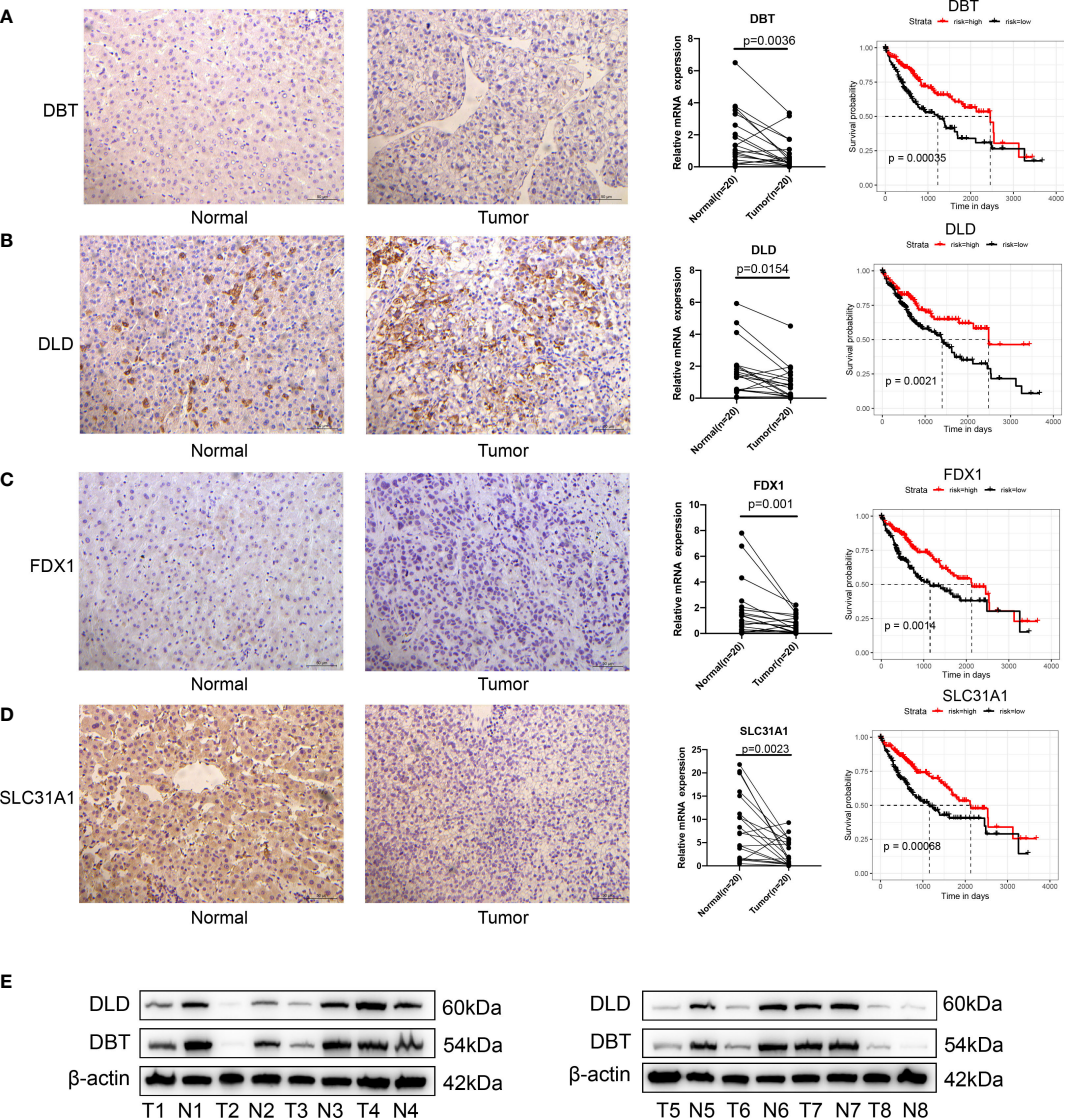
Validation of cuproptosis-related genes in HCC. **(A–D)** Immunohistology of DBT, DLD, FDX1 and SLC31A1 in normal and tumor tissues; qRT-PCR showed that compared with the adjacent normal tissues, FDX1, DBT, DLD and SLC31A1 were significantly expressed lower in tumor tissues; results of the KM analysis showing expressions of each cuproptosis-related gene that significantly influenced the survival of TCGA. Red line represents the high-risk group and dark line the low-risk group. **(E)** the expression of DLD, DBT by Western blot in 8 paired tissue samples, with differences between HCC and normal tissues.

A transcriptional profile of cuproptosis-related genes in HCC was then constructed. PPI enrichment analysis showed that DBT, GCSH, LIPT1, DLAT, PDHA1, PDHB, DLD and DLST comprised the main components of MCODE, which involved glyoxylate metabolism and glycine degradation, Metabolism of amino acids and derivatives along with acetyl-CoA metabolic processes are summarized in **Figure 1E**. Results of the pathway enrichment analysis indicated that cuproptosis-related genes were also significantly enriched in biological pathways involved with copper ion import and protein lipoylation (**Figure 1F**). Moreover, gene expression analysis in bulk RNA-seq showed that the majority of cuproptosis-related genes, including ATP7B, DBT, DLD, DLST, FDX1, GCSH, LIAS, PDHA1, PDHB and SLC31A1, exhibited a relative lower expression level in HCC samples compared with the normal liver samples. ATP7A was the only signature which was expressed at significantly higher levels in HCC samples as compared with that observed in normal control samples (**Figure 1G**). We further explored the expression level of FDX1 in Pan-cancer, which showed that HCC tissues expressed higher FDX1 than the majority of cancer types (**Figure S1B**). Spearman correlation analysis was also included to reveal correlations among the expressions of these cuproptosis-related genes, as presented below and in **Figure 1H**. In addition, immunohistochemical technique was applied to explore the expression level of cuproptosis-related genes in HCC tissues (**Figures 2A–D**). We further verified the expression level of hubgene in clinical tissues. qRT-PCR showed that compared with the adjacent normal tissues, FDX1, DBT, DLD, SLC31A1 was significantly expressed lower in HCC tissues (**Figures 2A–D**; **S2A**). Subsequently, we validated the expression of DLD, DBT by Western blot in 16 paired tissue samples, with differences between HCC and normal tissues (**Figures 2E**; **S2C**).

### Cuproptosis-related genes greatly influence the prognosis of HCC

When evaluating the prognostic value of these 13 cuproptosis-related genes, expression levels of 9 genes, including FDX1, LIPT1, LIAS, DLD, DLAT, DBT, SLC31A1, ATP7A and ATP7B, were significantly associated with the prognosis of human HCC based on TCGA-LIHC data (**Figures 2A-D**; **S2B**). Specifically, high expressions of FDX1, LIAS, DLD, DBT, SLC31A1 and ATP7B suggested a better prognosis while high expressions of LIPT1, DLAT and ATP7B suggested poor outcomes. Expression levels of the remaining four genes, PDHA1, PDHB, GCSH and DLST appeared to have no significant effect on prognosis (**Figure S2B**).

### Single cell analysis identified cuproptosis-related hepatocytes

To better understand cuproptosis at the single cell level, we retrieved and performed a single cell analysis of a human HCC dataset, which contained 14 pairs of human HCC tumor samples using the 10X genomics platform (**Table S1**).

**Figure 3:**
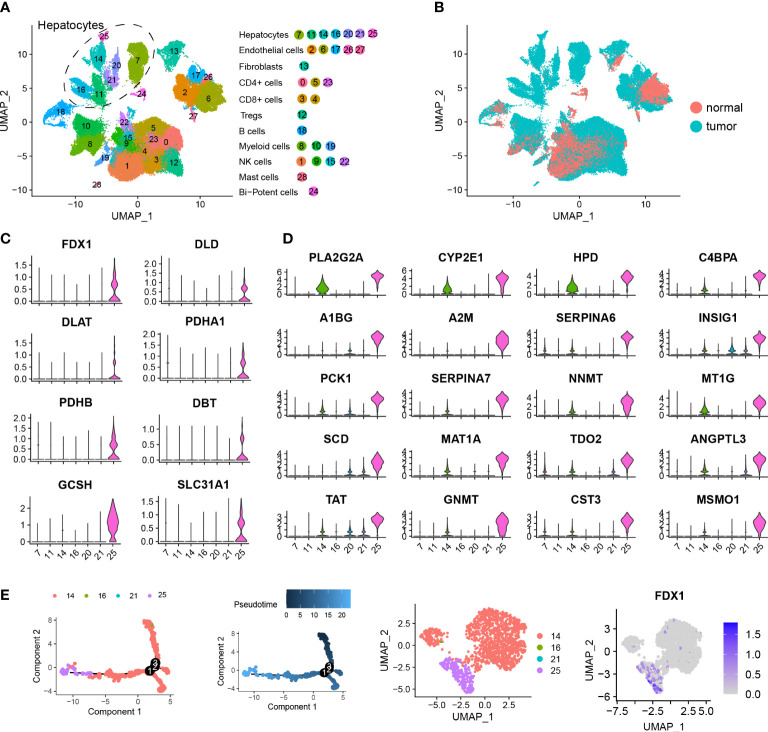
Single cell analysis identifying cuproptosis-related hepatocytes. **(A)** UMAP visualization showing the hepatocellular carcinoma landscape of 73,589 cells containing 29 clusters across 28 HCC samples using dotplot. Each dot represents a single cell and hepatocyte subgroups are encircled with dotted lines. **(B)** UMAP visualization showing the distribution of the single cell HCC landscape between tumor and normal tissues. **(C)** Violin plots showing expression levels of 8 cuproptosis-related genes among 7 hepatocyte clusters, including FDX1, DLD, DLAT, PDHA1, PDHB, DBT, GCSH and SLC31A1. **(D)** Violin plots showing expression levels of the top 20 marker genes in cuproptosis-related hepatocytes. **(E)** Pseudotime analysis showing the predicted evolution of hepatocytes. UMAP visualization showing the distribution of hepatocytes and gene expression of FDX1.

After filtering low-quality cells, we obtained 73,589 cells and performed a downstream analysis including normalization, scaling, dimension reduction and clustering. Following detailed annotations using canonical markers, we constructed a cellular landscape of human HCC (**Figure 3A**). Those cells (numbers, percent) exhibiting a distinct distribution between tumor and normal tissues are contained in **Figure 3B**, consisting of Hepatocytes (12,646, 17.18%), Endothelial cells (11,207, 15.22 cells), Fibroblasts (1,891, 2.56%), CD4+ T cells (14,136, 19.21%), CD8+ T cells (9,316, 12.66%), Treg (2,866, 3.89%), B cells (1,376, 1.87%), Myeloid cells (6,726, 9.14%), NK cells (12,970, 17.62%), Mast cells (119, 0.2%) and Bi-potent cells (336, 0.5%).

A total of 12,646 hepatocytes across 7 clusters were identified. As based on the expression of cuproptosis-related genes, we found cluster 25 was distinct and labeled it as a cuproptosis-related hepatocyte. This cuproptosis-related hepatocyte highly expressed a number of cuproptosis-related genes including FDX1, DLD, DLAT, PDHA1, PDHB, DBT, GCSH and SLC31A1 (**Figure 3C**), which were mostly located in tumor tissues (**Figure 3B**). To better understand and describe the characterization of cuproptosis-related hepatocytes, we performed a differentially expressed analysis and identified a list of gene signatures (**Table S4**). The top 20 marker genes are shown in **Figure 3D**, and include PLA2G2A, CYP2E1, HPD, C4BPA, A1BG, A2M, SERPINA6, INSIG1, PCK1, SERPINA7, NNMT, MT1G, SCD, MAT1A, TDO2, ANGPTL3, TAT, GNMT, CST3 and MSMO1. 319 DEGs satisfying the criteria for filtration (|logFC| > 1.5 and adj.p.value < 0.5) were further subjected to GO and KEGG enrichment analysis (**Table S5**). Results of this analysis indicated that these DEGs were involved in pathways of RNA catabolic process, translational initiation, protein targeting and localization to endoplasmic reticulum (**Figures S3A, B**). To further assess the evolution process of cuproptosis-related hepatocytes, a pseudotime analysis for a typical sample named P28 was performed, with the findings that cuproptosis-related hepatocytes were located at an advanced stage of evolution (**Figure 3E**).

### Identification of cuproptosis patterns mediated by the 13 identified gene signatures

**Figure 4:**
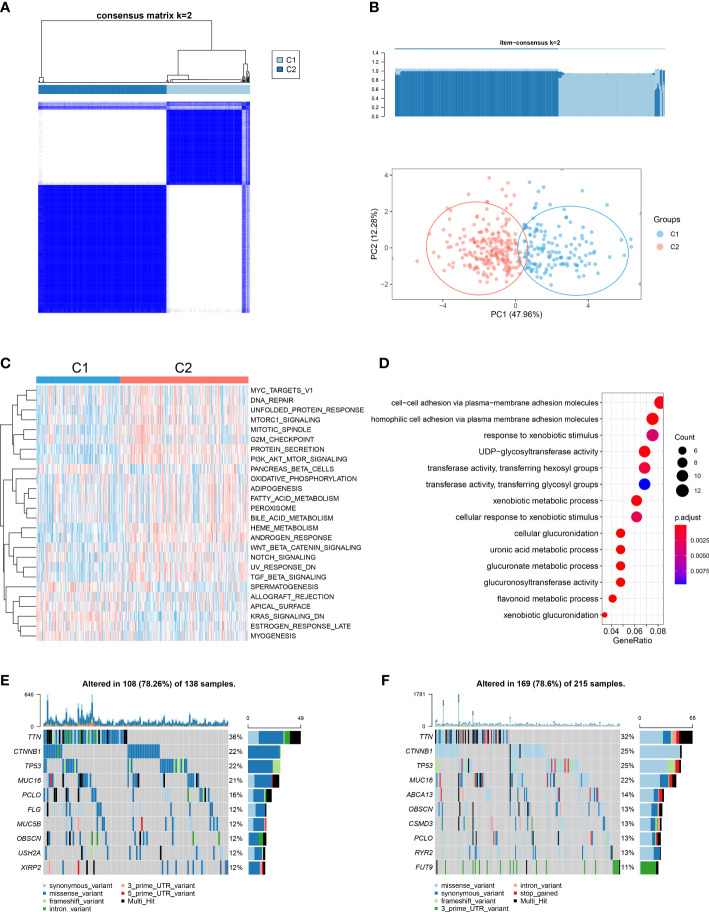
Cuproptosis patterns in the TCGA-LIHC cohort and relevant biological pathways. **(A)** Similarity matrix of TCGA-LIHC patients derived from consensus clustering assays. **(B)** Principal component analysis results for the two distinct patterns in the TCGA-LIHC cohort. **(C)** GSVA scores of representative Hallmark pathways in cuproptosis-C1 and cuproptosis-C2 patterns as shown in the heatmap. **(D)** Dotplot showing GO term enrichment analysis of DEGs between patterns. **(E)** Of the 138 patients in cuproptosis-C1, 108 (78.26%) showed gene mutations. The right bar plot shows the mutation frequency of each gene and each column represents an individual patient. **(F)** Of the 215 patients in cuproptosis-C2, 169 (78. 6%) showed gene mutations.

We noted that cuproptosis could play an important role in HCC, *via* aspects involving genomics, transcriptomics and proteomics. Accordingly, we performed a consensus clustering analysis using the ConsensusClusterPlus function to classify samples with differing cuproptosis patterns based on expressions of the 13 gene signatures identified. Two distinct pattern clusters were found, consisting of 144 samples in cluster1 (cuproptosis-C1) and 221 samples in cluster2 (cuproptosis-C2) (**Figures 4A, B**). We also observed a significant difference in the expression of cuproptosis-related genes between these two cuproptosis patterns. Specifically, all genes were significantly elevated in the cuproptosis-C2 subtype (**Figure S4A**), which suggested its potential impact as a prognosis marker. Although the results of our KM survival analysis failed to achieve statistical significance, the direction of these results showed a trend for cuproptosis-C2 to be associated with a poor prognosis (**Figure S4B**).

To examine the potential for biological differences between these two cuproptosis patterns, we conducted a pathway enrichment analysis using GSVA and GO functions. Results from GSVA indicated that cuproptosis-C2 was significantly enriched in more pathways than cuproptosis-C1 (**Figure 4C**; **Table S6**). Moreover, cuproptosis-C2 was found to be associated with pathways involved with tumorigenesis and development (MYC targets v1, mTORC1 signaling, PI3K/AKT/mTOR signaling and Wnt/β-catenin signaling) as well as pathways involved with metabolism (adipogenesis, fatty acid metabolism, bile acid metabolism and PEROXISOME). In contrast, cuproptosis-C1 was substantially less involved with such pathways, only showing high levels of KRAS signaling related knockdown genes. Top 3 GO terms enriched in DEGs between these two patterns were cellular adhesion *via* plasma membrane adhesion molecules, responses to xenobiotic stimuli and UDP glycosyltransferase activity (**Figure 4D**).

We also compared the mutation profilers of these two cuproptosis patterns. Cuproptosis-C1 showed 108 alterations within 138 samples (78.26%) and cuproptosis-C2 169 alterations within 215 samples (78.6%) (**Figures 4E, F**). Two cuproptosis patterns shared identical mutation genes for the top genes including TTN, CTNNB1, TP53, MUC16, PCLO and OBSCN. Top unique gene mutations observed between these two patterns consisted of FLG, MUC5B, USH2A and XIRP2 for cuproptosis-C1 and ABCA13, CSMD3, RYR2 and FUT9 for cuproptosis-C2. The same top 5 mutation effects present in these two patterns included missense variant, synonymous variant, 3’ UTR variant, intron variant and frameshift variant.

### Cuproptosis patterns as characterized by different immune profilers

**Figure 5:**
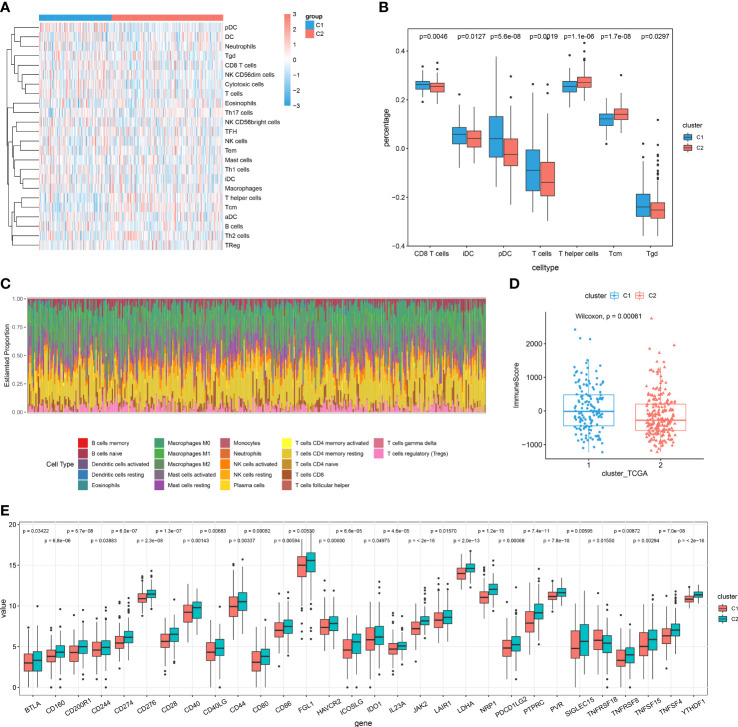
Cuproptosis patterns as characterized by different immune profilers. **(A)** Enrichment levels of 24 immune-related cells in the cuproptosis-C1 and cuproptosis-C2 subtypes using ssGSEA. **(B)** Boxplots showing differences in cell type percent between immune patterns. **(C)** Histograms displaying the proportion of 22 different types of immune cells in patterns as based on CIBERSORT. **(D)** Boxplots showing estimated differences in immune scores. **(E)** Gene expression levels of 20 immune checkpoints between the two patterns.

A landscape was generated using heatmap with the ssGSEA technique. In this way, it was possible to visualize and describe differences in relative immune infiltration of the 24 immune cell types between cuproptosis patterns showing distinct immune patterns (**Figure 5A**). CD8+ T, Tgd, iDC and pDC cells were mainly enriched in the cuproptosis-C1 subtype, identified as the cytotoxic immune patterns (**Figure 5B**), whereas, T helper and Tcm cells showed relatively higher proportions in the cuproptosis-C2 subtype, labeled as the regulatory immune patterns. CIBERSORT, a deconvolution method to indicate immune cells in TME, was also used to evaluate immune infiltration profilers with the results of this assay demonstrating relatively consistent results as described above (**Figure 5C**). When assessing the results of immune and stromal scores in these two patterns, cluster 1 showed higher immune score than cluster2 (**Figure 5D**), which was in accord with the results of immune infiltration (**Figure 5B**).

Next, we examined the expressions of immune checkpoint genes between these two patterns. Interestingly, expression levels of all significantly different immune checkpoints were mainly higher in the cuproptosis-C2 subtypes (**Figure 5E**). Results when evaluating the prognosis value of these 30 immune checkpoint genes revealed that 13 immune checkpoint genes significantly influenced the prognosis of HCC patients. In specific, high expressions of BTLA, CD28, CD40LG, CD244, ICOSLG, IL23A, PDCD1LG2 and TNFRSF8 were associated with a better prognosis (**Figure S5A**), while high expressions of CD40, LAIR1, LDHA, TNFSF4 and YTHDF1 indicated poor outcomes (**Figure S5B**). To further predict the efficacy of immune checkpoint therapy, we applied TIDE analysis to two patterns, which showed that cuproptosis-C1 indicated relative higher score of CD8 and Merck18 (**Figure S5C**), in which the later can contribute to T-cell dysfunction. And cuproptosis-C1 significantly exhibited dysfunction for immune checkpoint therapy (**Figure S5C**).

### Cuproptosis and phenotype-related DEGs in HCC

We further evaluated the potential for cuproptosis-related transcriptional changes between these two cuproptosis patterns in HCC. A total of 169 DEGs were identified as based on the criteria consisting of adj.P.Val<0.05 and abs(logFC)>1.5 (**Table S7**). Top 3 GO terms enriched in DEGs between the two patterns were cellular adhesion *via* plasma membrane adhesion molecules, responses to xenobiotic stimuli and UDP glycosyltransferase activity (**Figure 4D**).

### Construction and validation of a prognosis model as based on DEGs in HCC

**Figure 6:**
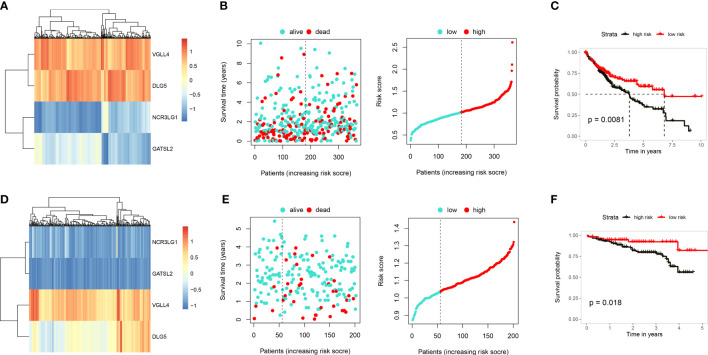
Construction and validation of a cuproptosis prognosis model. **(A)** Heatmap showing gene expressions in the cuproptosis prognosis model including, VGLL4, DLG5, NCR3LG1 and GATSL2 in TCGA-LIHC. **(B)** Distribution of risk scores and survival status of TCGA-LIHC. **(C)** Results of KM analysis indicating that the prognosis model significantly influenced survival of patients in TCGA. Dark line represents the high-risk group and red line the low-risk group. **(D)** Heatmap showing gene expressions of the cuproptosis prognosis model in ICGC. **(E)** Distribution of risk scores and survival status of ICGC. **(F)** Results of KM analysis indicating that the prognosis model significantly influenced survival of patients in TCGA. Dark line represents the high-risk group and red line the low-risk group.

A univariate Cox regression analysis was conducted by combining expression levels of the 169 intersecting genes and survival data from 421 samples. 13 genes proven to be closely related to prognosis, including RECQL, SOX6, RAB23, SMC4, APAF1, IGF2BP3, VGLL4, ITGB1, DLG5, ADAM17, UGT1A6, NCR3LG1 and GATSL2 (**Figure S6**). A multivariate regression analysis was subsequently performed which identified 4 gene signatures, VGLL4, DLG5, NCR3LG1 and GATSL2, with VGLL4 and DLG5 demonstrating relatively higher levels of expression than NCR3LG1 and GATSL2 in the TCGA-LIHC data (**Figure 6A**). Based on the expression profiles and coefficients of the four genes, the cuproptosis score = (0.3343)*VGLL4 + (-0.1598)*DLG5 + (-0.0808) *NCR3LG1 and + (-0.1649)* GATSL2. Then, based on these risk score, we divided LIHC patients into either a high- or low-risk group. Results from the Kaplan-Meier survival analysis substantiated that the low-risk group showed a significantly better prognosis (P=0.0081; **Figures 6B, C**). When validating the 4 gene signature prognosis-model using an independent cohort, ICGC LICA-FR (**Figures 6D–F**), significantly better outcomes were obtained with the low-risk group (p=0.018). The above findings substantiated the robust prognostic ability of the cuproptosis score in HCC.

### Establishment of a cuproptosis index and evaluation of its clinical relevance

**Figure 7:**
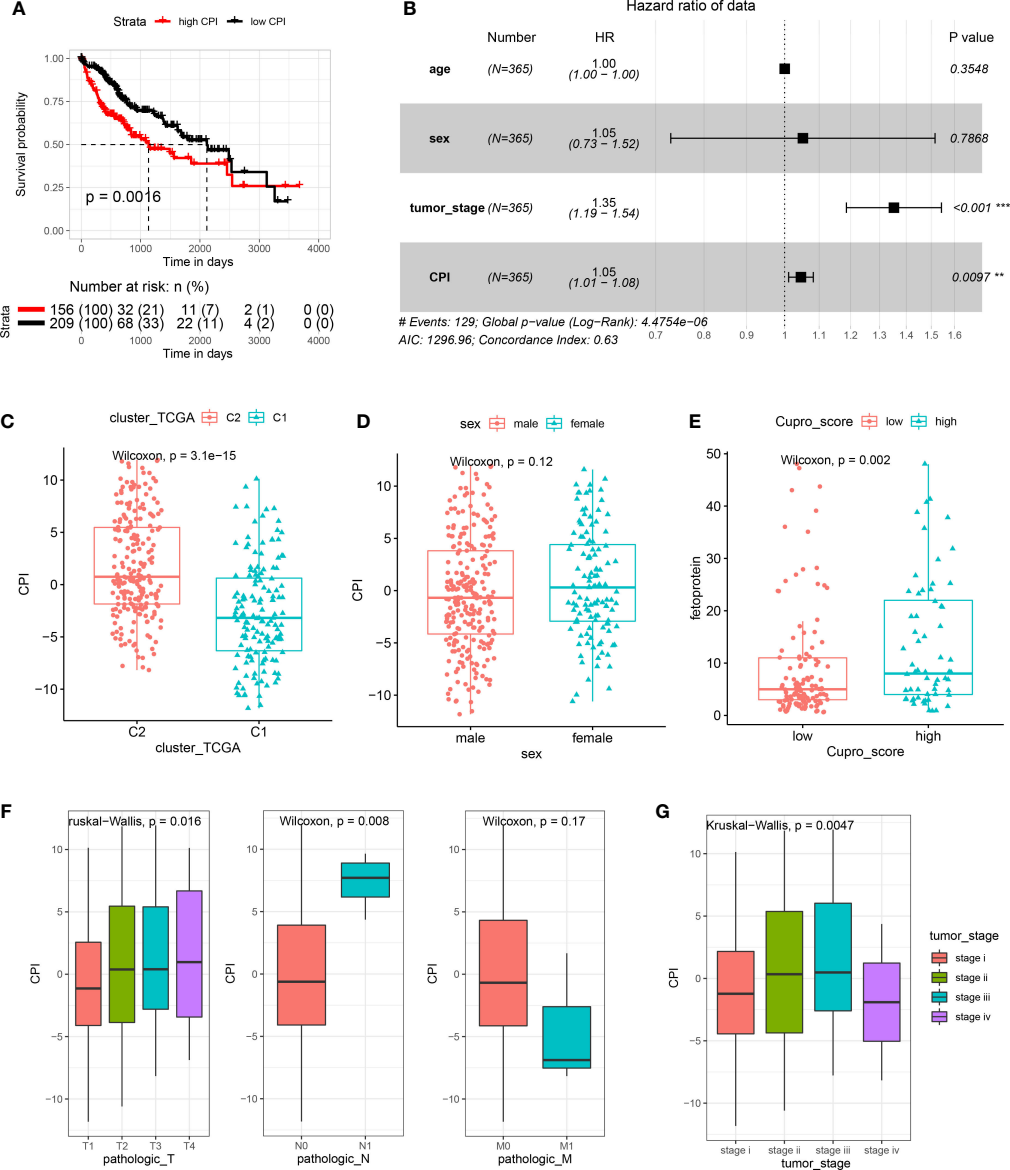
Establishment and evaluation of the cuproptosis index (CPI). **(A)** Results of KM analysis indicating that the CPI significantly influenced the survival of patients in TCGA. Red line represents the high-risk group and dark line the low-risk group. **(B)** Results of the multivariate analysis based on TCGA-LIHC. **(C)** Boxplots showing cuproptosis-C2 was associated with high CPI scores. **(D)** CPI scores failed to show a statistically significant difference between genders. **(E)** High CPI scores were associated with high levels of alpha fetoprotein. **(F)** Boxplots showing that TNM stage was associated with distinct CPI scores. **(G)** Advanced tumor stage was related with high CPI scores.

We developed a score scheme termed the cuproptosis index (CPI). The CPI was based on cuproptosis pattern related genes, which could then be used to quantify cuproptosis patterns within HCC patients (**Table S8**). A randomforest algorithm was used to identify 11 gene signatures to generate a high accuracy index system (**Figure S7A**). Among 365 tumor samples within TCGA-LIHC, we isolated 156 samples with high CPI values and 209 samples with low CPI values. Results from the survival analysis indicated that the high CPI group exhibited significantly poorer outcomes (**Figure 7A**). To further clarify whether CPI is an independent risk factor for the prognosis of HCC patients, we conducted multivariate analyses. The results indicated that CPI acts as an independent factor in the prediction of prognosis of HCC patients (p < 0.05, HR > 1, **Figure 7B**). When assessing the relationship between CPI and cuproptosis immune patterns, we found that the cuproptosis-C2 subtype was linked with higher CPI score, whereas the cuproptosis-C1 subtype exhibited lower CPI score (**Figure 7C**). We then evaluated the clinical relevance of CPI, with the results of these analyses indicating no gender differences in CPI scores (**Figure 7D**) however, higher CPI scores were associated with significantly increased levels of AFP (**Figure 7E**) and were correlated with an advanced tumor status (**Figures 7F, G**) in HCC patients. CPI performance was subsequently validated in an independent ICGC cohort (**Figure S7**).

**Figure 8:**
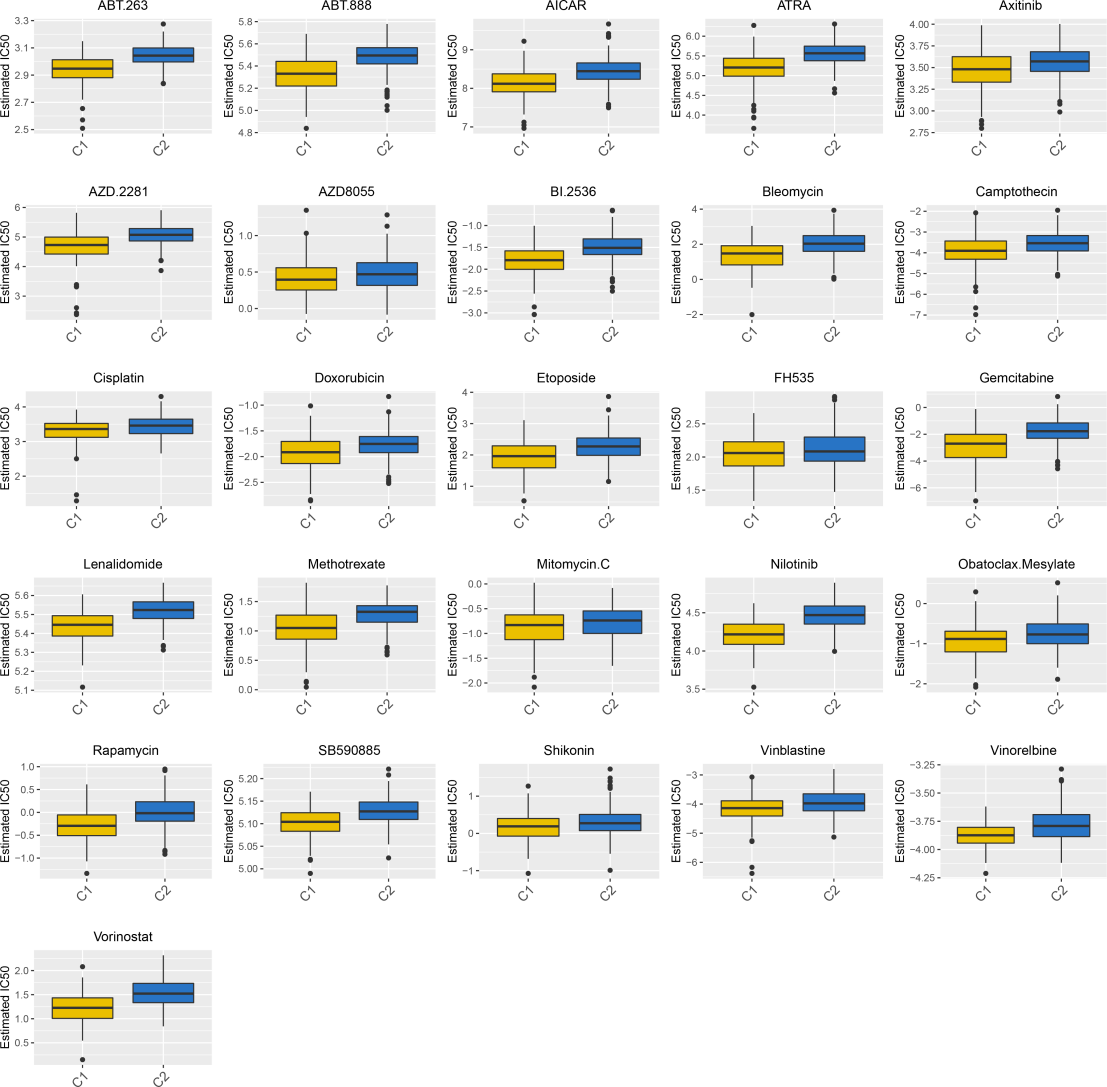
A total of 26 potential therapeutic drugs in HCC with differential IC50 based high- and low-CPI groups.

To explore the therapeutic potential drugs based on high- and low-CPI groups, we compiled 26 drugs from prior studies that had been tested and reported to have therapeutic promise for HCC, especially including All-trans retinoic acid (ATRA), Axitinib, AZD.2281 (Olaparib), AZD8055, Camptothecin, Doxorubicin, Gemcitabine, Rapamycin, Cisplatin, Bleomycin, Methotrexate and Mitomycin.C (MMC). The sensitivity to the aforementioned 26 drugs in high- or low-risk groups was predicted using the “pRRophetic” algorithm. The low-CPI group got higher estimated IC50 values than the high-CPI group, and this finding suggested that a greater CPI could predict increased sensitivity to these therapeutic drugs in HCC patients (**Figure 8**).

## Discussion

As a novel mean of cell death, copper-triggered cuproptosis, was found to involve mitochondrial cell death, and is independent of other known cell death processes, including apoptosis (10), necroptosis (11), pyroptosis (12) and ferroptosis (13). The novelty, uniqueness and relatively limited information on cuproptosis in the literature stimulated our interests into investigating its mechanisms and potential value for the diagnosis and treatment of HCC. Therefore, we integrated the available multi-omics data to elucidate the potential function of cuproptosis in HCC.

The most significant contribution of this study was the characterization and evaluation of cuproptosis as related to HCC. Three key points were generated from this study: 1) We depicted the genomic and transcriptome patterns of cuproptosis in HCC and found cuproptosis-related hepatocytes at the single cell level, 2) We identified two immune patterns as based on the expressions of cuproptosis-related genes and 3) We constructed and validated a prognosis model and cuproptosis index based on DEGs of cuproptosis patterns.

To achieve these findings, we first assessed the mutations in cuproptosis-related genes in HCC. Missense variants, 5’ UTR variants and synonymous variants were the top 3 mutation phenotypes, with the highest frequency variant being ATP7A, followed by PDHA1 and DLST. As a copper exporter, a mutation of ATP7A results in Wilson’s disease, however studies relating ATP7A mutations with HCC are rare (34). Moreover, CNV alterations were also prevalent in the 13 cuproptosis-related genes we identified. We then established the transcriptomic profilers for these 13 cuproptosis-related genes. Results of our pathway enrichment analysis showed that high levels of copper ion import, protein lipoylation and cellular amino acid metabolic process were present, which was in line with findings of a previous report indicating that cuproptosis functions by binding of copper in the TCA cycle and produces lipoylated protein aggregation and protein loss (8). While the potential value of these cuproptosis-related genes for the prognosis of HCC patients remains largely unknown, we were somewhat surprised to find that most of these genes (76.92%) showed differential expressions between tumor versus normal tissues, and more than half were prevalent in normal tissues. Nine genes, including FDX1, LIPT1, LIAS, DLD, DLAT, DBT, SLC31A1, ATP7A and ATP7B, were significantly corelated with overall survival, which indicated that cuproptosis clearly impacted the survival of hepatocytes in HCC. Compared with bulk RNA-seq, single cell RNA-seq exhibited a higher resolution which then enabled the generation of more detailed information and the identification of novel cell clusters (35). As a result, it was now possible to evaluate the single cell data within the HCC cohort. After delineating the HCC landscape and detailing cell annotations, we identified a cluster which was strongly related with cuproptosis. We named this cluster, cuproptosis-related hepatocytes based on the expression of the cuproptosis-related genes. We found that the unique genes present in this cluster were involved with a number of pathways, such as RNA catabolic process, protein targeting and localization to endoplasmic reticulum. Further, pseudotime analysis revealed that this cluster was present at the advanced stages of HCC evolution. Accordingly, these results strongly implicated a potential role for cuproptosis in HCC.

We identified two distinct cuproptosis patterns characterized by different immune phenotypes. Cuproptosis-C1 was characterized as a cytotoxic immune phenotype while cuproptosis-C2 as a regulatory immune pattern, which was corelated with diverse pathways involving tumorigenesis, tumor development and molecular metabolism. It has been reported that tumor immune infiltration plays a major role in tumor progression and immunotherapeutic efficacy for HCC (36, 37). Baseline levels of tumor-infiltrating CD4+ T cells, inflammatory cytokines and immune checkpoints have all been turned out to be correlated with the likelihood of an immune response. We also found that cuproptosis-C2 showed high expression levels associated with immune checkpoints, including PD-L1, PD-L2 and PVR. To further predict the efficacy of immune checkpoint therapy, we applied TIDE analysis to two patterns, which showed that cuproptosis-C2 indicated prevalent efficacy of immune checkpoint inhibitors therapy. Those findings suggest a potential response value for an immunotherapeutic benefit.

Functional pathways enriched by DEGs, as identified between the two immune patterns, implicated pathways that included cellular adhesion *via* plasma membrane adhesion molecules, responses to xenobiotic stimuli and UDP glycosyltransferase activity. Based on these DEGs, we established a prognostic model containing VGLL4, DLG5, NCR3LG1 and GATSL2, which was further validated in an independent cohort. VGLL4 can inhibit cell proliferation and tumor growth in HCC (38), which was attributable to an arrest of the G2/M phase and apoptosis promotion by adenovirus (39). DLG5 was recently identified to be a novel tumor related gene in pituitary tumors as based on single cell data (40), and has also been shown to be associated with overall survival in HCC (41). NCR3LG1 functions to encode the ligand of the natural cytotoxicity receptor NKp30 and knockdown of NCR3LG1 protects against cell death in the human chronic-myelogenous-leukemia (CML) cell line (42). Finally, GATSL2 has been reported to be involved with gene fusions in the malignant progression of spinal cord gliomas (43).

The importance of establishing cell death models and scores as a means to guide tumor treatments has been described in previous studies (44, 45). Therefore, we developed a score scheme termed the cuproptosis index (CPI) to quantify cuproptosis patterns. Survival analysis results showed that high CPI scores were associated significantly poorer outcomes. These findings suggest that cuproptosis plays an important role in cell death related to HCC and was linked to the cuproptosis-C2 subtype, which exhibits better immune responses. Intriguingly, a positive correlation exists between CPI and AFP, and the latter is a biomarker for tumor genesis and cancer progression in HCC (46). To the best of our knowledge, this CPI represents the first cuproptosis-related score scheme to quantify HCC, and warrants further investigation to substantiate its value.

Our study has limitations. Although we reviewed studies associated with cuproptosis and curated a list of 13 cuproptosis-related genes, a series of newly identified gene signatures will need to be evaluated and integrated into the model to corroborate the value of these cuproptosis patterns. The cuproptosis-related hepatocytes found in HCC were based on single cell data, therefore their function and performance need to examine using *in vivo* models. Finally, the value of the CPI will need to be evaluated in additional cohorts.

Taken together, in this study we have performed a comprehensive evaluation of cuproptosis as related to HCC using multi-Omics. Further clinical and basic studies with HCC using multiple techniques will be required to substantiate our findings regarding the importance and role of cuproptosis in HCC.

## Data availability statement

The original contributions presented in the study are included in the article/**Supplementary Material**. Further inquiries can be directed to the corresponding authors.

## Ethics statement

The studies involving human participants were reviewed and approved by Affiliated Hospital of Qingdao University. The patients/participants provided their written informed consent to participate in this study. Written informed consent was obtained from the individual(s) for the publication of any potentially identifiable images or data included in this article.

## Author contributions

JC, SL and XL contributed to the research design. XL, RL and BW contributed to the data management and statistical analyses. PJ performed the basic experiments including western blot, qRT-PCR and immunohistochemical staining. JC, RL, XL and KZ wrote the manuscript. All authors contributed to the article and approved the submitted version.

## Funding

This work was supported by the National Natural Science Foundation of China (No. 81670600).

## Acknowledgments

The authors would like to thank the Organ Transplantation Center and the Key Laboratory of Organ Transplantation of Affiliated Hospital of Qingdao University for their technical support. We thank Pro. Tao Shan (Qingdao University) and all the members of his anatomy research team for support. We also thank Dr. Jianming Zeng (University of Macau), and all the members of his bioinformatics team, biotrainee, for generously sharing their experience and codes.

## Conflict of interest 

The reviewer WZ declared a shared parent affiliation with the authors XL, BW, KZ, JC to the handling editor at the time of review.

The remaining authors declare that the research was conducted in the absence of any commercial or financial relationships that could be constructed as a potential conflict of interest.

## Publisher’s note

All claims expressed in this article are solely those of the authors and do not necessarily represent those of their affiliated organizations, or those of the publisher, the editors and the reviewers. Any product that may be evaluated in this article, or claim that may be made by its manufacturer, is not guaranteed or endorsed by the publisher.

## Supplementary material

Supplementary Figure 1 Workflow of the study. **(A)** Route diagram showing the main flow of the study, including multi-omics characterizations of cuproptosis-related genes, construction of immune patterns based on cuproptosis-related genes, identification of prognostic gene signatures as based on DEGs and establishment and evaluation of the cuproptosis index. **(B)** Expression patterns of FDX1 in pan-cancer. (* p < 0.05; ** p < 0.01; *** p < 0.001).

Supplementary Figure 2 QRT-PCR and survival analysis of cuproptosis-related genes in HCC. **(A)** qRT-PCR showed that compared with the adjacent normal tissues, FDX1, DBT, DLD and SLC31A1 were lowly expressed in tumor tissues. **(B)** Results of the KM analysis showing expressions of each cuproptosis-related gene that influenced the survival of TCGA. Red line represents the high-risk group and dark line the low-risk group. **(C)** the expression of DLD, DBT by Western blot in other 8 paired tissue samples, with differences between HCC and normal tissues.

Supplementary Figure 3 Functional enrichment of cuproptosis-related hepatocytes. **(A)** Dot plots showing the top 10 GO terms enriched by DEGs in cuproptosis-related hepatocytes. **(B)** Dot plots showing the top 10 KEGG pathways enriched by DEGs in cuproptosis-related hepatocytes.

Supplementary Figure 4 Gene expression and survival analysis of cuproptosis patterns. **(A)** Boxplots showing the gene expression of 13 cuproptosis-related genes between the two cuproptosis patterns. (* p < 0.05; ** p < 0.01; *** p < 0.001). **(B)** Results of KM analysis showing survival probabilities for patients in cuproptosis-C1 and cuproptosis-C2 groups. Yellow line represents the Cuproptosis-C1 group and blue line the Cuproptosis-C2 group.

Supplementary Figure 5 Survival analysis of immune checkpoint genes. **(A)** Results of KM analysis showing high expressions of genes that were associated with a better prognosis: BTLA, CD28, CD40LG, CD244, ICOSLG, IL23A, PDCD1LG2 and TNFRSF8. Red line represents the high-risk group and dark line the low-risk group. **(B)** Results of KM analysis showing high expressions of genes associated with poor outcomes: CD40, LAIR1, LDHA, TNFSF4, YTHDF1. Red line represents the high-risk group and dark line the low-risk group. **(C)** Boxplots showing Merck18, CD8 and Dysfunction scores between two patterns.

Supplementary Figure 6 Univariate cox analysis of DEGs by patterns. Forest plot showing results of univariate Cox regression of the 13 genes. The 95% confidence interval for each group was indicated by the length of the horizontal line. Hazard ratio (HR) of all patients was indicated by the vertical dotted line.

Supplementary Figure 7 Validation and evaluation of the cuproptosis index (CPI). **(A)** Line chart showing 11 genes exhibiting top accuracy for CPI using cross validation. **(B)** Results of KM analysis showing that CPI scores significantly influenced the survival of patients in ICGC. Red line represents the high-risk group and dark line the low-risk group. **(C)** CPI scores failed to indicate a statistically significant difference between genders. **(D)** Advanced tumor stage was related with high CPI scores.

## References

[1] Chidambaranathan-Reghupaty S Fisher PB Sarkar D . Hepatocellular carcinoma (HCC): Epidemiology, etiology and molecular classification. Adv Cancer Res (2021) 149:1–61. doi: 10.1016/bs.acr.2020.10.001 33579421 PMC8796122

[2] Sung H Ferlay J Siegel RL Laversanne M Soerjomataram I Jemal A . Global cancer statistics 2020: GLOBOCAN estimates of incidence and mortality worldwide for 36 cancers in 185 countries. CA Cancer J Clin (2021) 71(3):209–49. doi: 10.3322/caac.21660 33538338

[3] Foerster F Gairing SJ Muller L Galle PR . NAFLD-driven HCC: Safety and efficacy of current and emerging treatment options. J Hepatol (2022) 76(2):446–57. doi: 10.1016/j.jhep.2021.09.007 34555422

[4] Llovet JM De Baere T Kulik L Haber PK Greten TF Meyer T . Locoregional therapies in the era of molecular and immune treatments for hepatocellular carcinoma. Nat Rev Gastroenterol Hepatol (2021) 18(5):293–313. doi: 10.1038/s41575-020-00395-0 33510460

[5] Ho DW Tsui YM Chan LK Sze KM Zhang X Cheu JW . Single-cell RNA sequencing shows the immunosuppressive landscape and tumor heterogeneity of HBV-associated hepatocellular carcinoma. Nat Commun (2021) 12(1):3684. doi: 10.1038/s41467-021-24010-1 34140495 PMC8211687

[6] Zhang G He J Ye X Zhu J Hu X Shen M . Beta-thujaplicin induces autophagic cell death, apoptosis, and cell cycle arrest through ROS-mediated akt and p38/ERK MAPK signaling in human hepatocellular carcinoma. Cell Death Dis (2019) 10(4):255. doi: 10.1038/s41419-019-1492-6 30874538 PMC6420571

[7] Yu Z Guo J Hu M Gao Y Huang L . Icaritin exacerbates mitophagy and synergizes with doxorubicin to induce immunogenic cell death in hepatocellular carcinoma. ACS Nano (2020) 14(4):4816–28. doi: 10.1021/acsnano.0c00708 32188241

[8] Tsvetkov P Coy S Petrova B Dreishpoon M Verma A Abdusamad M . Copper induces cell death by targeting lipoylated TCA cycle proteins. Science (2022) 375(6586):1254–61. doi: 10.1126/science.abf0529 PMC9273333 35298263

[9] Tang D Chen X Kroemer G . Cuproptosis: A copper-triggered modality of mitochondrial cell death. Cell Res (2022) 32(5):417–8. doi: 10.1038/s41422-022-00653-7 PMC9061796 35354936

[10] Carneiro BA El-Deiry WS . Targeting apoptosis in cancer therapy. Nat Rev Clin Oncol (2020) 17(7):395–417. doi: 10.1038/s41571-020-0341-y 32203277 PMC8211386

[11] Weinlich R Oberst A Beere HM Green DR . Necroptosis in development, inflammation and disease. Nat Rev Mol Cell Biol (2017) 18(2):127–36. doi: 10.1038/nrm.2016.149 27999438

[12] Bergsbaken T Fink SL Cookson BT . Pyroptosis: host cell death and inflammation. Nat Rev Microbiol (2009) 7(2):99–109. doi: 10.1038/nrmicro2070 19148178 PMC2910423

[13] Dixon SJ Lemberg KM Lamprecht MR Skouta R Zaitsev EM Gleason CE . Ferroptosis: An iron-dependent form of nonapoptotic cell death. Cell (2012) 149(5):1060–72. doi: 10.1016/j.cell.2012.03.042 PMC3367386 22632970

[14] Wang Y Zhang L Zhou F . Cuproptosis: A new form of programmed cell death. Cell Mol Immunol (2022) 19:867–8. doi: 10.1038/s41423-022-00866-1 PMC9338229 35459854

[15] Tsvetkov P Detappe A Cai K Keys HR Brune Z Ying W . Mitochondrial metabolism promotes adaptation to proteotoxic stress. Nat Chem Biol (2019) 15(7):681–9. doi: 10.1038/s41589-019-0291-9 PMC8183600 31133756

[16] Cobine PA Moore SA Leary SC . Getting out what you put in: Copper in mitochondria and its impacts on human disease. Biochim Biophys Acta Mol Cell Res (2021) 1868(1):118867. doi: 10.1016/j.bbamcr.2020.118867 32979421 PMC7680424

[17] Sharma A Seow JJW Dutertre CA Pai R Bleriot C Mishra A . Onco-fetal reprogramming of endothelial cells drives immunosuppressive macrophages in hepatocellular carcinoma. Cell (2020) 183(2):377–394.e21. doi: 10.1016/j.cell.2020.08.040 32976798

[18] Mayakonda A Lin DC Assenov Y Plass C Koeffler HP . Maftools: efficient and comprehensive analysis of somatic variants in cancer. Genome Res (2018) 28(11):1747–56. doi: 10.1101/gr.239244.118 PMC6211645 30341162

[19] Zhang H Meltzer P Davis S . RCircos: An r package for circos 2D track plots. BMC Bioinf (2013) 14:244. doi: 10.1186/1471-2105-14-244 PMC3765848 23937229

[20] Zhou Y Zhou B Pache L Chang M Khodabakhshi AH Tanaseichuk O . Metascape provides a biologist-oriented resource for the analysis of systems-level datasets. Nat Commun (2019) 10(1):1523. doi: 10.1038/s41467-019-09234-6 30944313 PMC6447622

[21] Yu G Wang LG Han Y He QY . clusterProfiler: An r package for comparing biological themes among gene clusters. OMICS (2012) 16(5):284–7. doi: 10.1089/omi.2011.0118 PMC3339379 22455463

[22] Li X Li S Wu B Xu Q Teng D Yang T . Landscape of immune cells heterogeneity in liver transplantation by single-cell RNA sequencing analysis. Front Immunol (2022) 13:890019. doi: 10.3389/fimmu.2022.890019 35619708 PMC9127089

[23] Stuart T Butler A Hoffman P Hafemeister C Papalexi E Mauck WM . Comprehensive integration of single-cell data. Cell (2019) 177(7):1888–1902.e21. doi: 10.1016/j.cell.2019.05.031 31178118 PMC6687398

[24] Aran D Looney AP Liu L Wu E Fong V Hsu A . Reference-based analysis of lung single-cell sequencing reveals a transitional profibrotic macrophage. Nat Immunol (2019) 20(2):163–72. doi: 10.1038/s41590-018-0276-y PMC6340744 30643263

[25] Trapnell C Cacchiarelli D Grimsby J Pokharel P Li S Morse M . The dynamics and regulators of cell fate decisions are revealed by pseudotemporal ordering of single cells. Nat Biotechnol (2014) 32(4):381–6. doi: 10.1038/nbt.2859 PMC4122333 24658644

[26] Wilkerson MD Hayes DN . ConsensusClusterPlus: a class discovery tool with confidence assessments and item tracking. Bioinformatics (2010) 26(12):1572–3. doi: 10.1093/bioinformatics/btq170 PMC2881355 20427518

[27] Hanzelmann S Castelo R Guinney J . GSVA: gene set variation analysis for microarray and RNA-seq data. BMC Bioinf (2013) 14:7. doi: 10.1186/1471-2105-14-7 PMC3618321 23323831

[28] Newman AM Liu CL Green MR Gentles AJ Feng W Xu Y . Robust enumeration of cell subsets from tissue expression profiles. Nat Methods (2015) 12(5):453–7. doi: 10.1038/nmeth.3337 PMC4739640 25822800

[29] Jiang P Gu S Pan D Fu J Sahu A Hu X . Signatures of T cell dysfunction and exclusion predict cancer immunotherapy response. Nat Med (2018) 24(10):1550–8. doi: 10.1038/s41591-018-0136-1 PMC6487502 30127393

[30] Zhang B Wu Q Li B Wang D Wang L Zhou YL . m(6)A regulator-mediated methylation modification patterns and tumor microenvironment infiltration characterization in gastric cancer. Mol Cancer (2020) 19(1):53. doi: 10.1186/s12943-020-01170-0 32164750 PMC7066851

[31] Chong W Shang L Liu J Fang Z Du F Wu H . m(6)A regulator-based methylation modification patterns characterized by distinct tumor microenvironment immune profiles in colon cancer. Theranostics (2021) 11(5):2201–17. doi: 10.7150/thno.52717 PMC7797678 33500720

[32] Yang W Soares J Greninger P Edelman EJ Lightfoot H Forbes S . Genomics of drug sensitivity in cancer (GDSC): A resource for therapeutic biomarker discovery in cancer cells. Nucleic Acids Res (2013) 41(Database issue):D955–61. doi: 10.1093/nar/gks1111 PMC3531057 23180760

[33] Li T Fu J Zeng Z Cohen D Li J Chen Q . TIMER2.0 for analysis of tumor-infiltrating immune cells. Nucleic Acids Res (2020) 48(W1):W509–14. doi: 10.1093/nar/gkaa407 PMC7319575 32442275

[34] Davis CI Gu X Kiefer RM Ralle M Gade TP Brady DC . Altered copper homeostasis underlies sensitivity of hepatocellular carcinoma to copper chelation. Metallomics (2020) 12(12):1995–2008. doi: 10.1039/d0mt00156b 33146201 PMC8315290

[35] Sun Y Wu L Zhong Y Zhou K Hou Y Wang Z . Single-cell landscape of the ecosystem in early-relapse hepatocellular carcinoma. Cell (2021) 184(2):404–421.e16. doi: 10.1016/j.cell.2020.11.041 33357445

[36] Ruf B Heinrich B Greten TF . Immunobiology and immunotherapy of HCC: spotlight on innate and innate-like immune cells. Cell Mol Immunol (2021) 18(1):112–27. doi: 10.1038/s41423-020-00572-w PMC7852696 33235387

[37] Llovet JM Castet F Heikenwalder M Maini MK Mazzaferro V Pinato DJ . Immunotherapies for hepatocellular carcinoma. Nat Rev Clin Oncol (2022) 19(3):151–72. doi: 10.1038/s41571-021-00573-2 34764464

[38] Guo Y Yao B Zhu Q Xiao Z Hu L Liu X . MicroRNA-301b-3p contributes to tumour growth of human hepatocellular carcinoma by repressing vestigial like family member 4. J Cell Mol Med (2019) 23(8):5037–47. doi: 10.1111/jcmm.14361 PMC6653225 31207037

[39] Xie W Hao J Zhang K Fang X Liu X . Adenovirus armed with VGLL4 selectively kills hepatocellular carcinoma with G2/M phase arrest and apoptosis promotion. Biochem Biophys Res Commun (2018) 503(4):2758–63. doi: 10.1016/j.bbrc.2018.08.036 30119884

[40] Cui Y Li C Jiang Z Zhang S Li Q Liu X . Single-cell transcriptome and genome analyses of pituitary neuroendocrine tumors. Neuro Oncol (2021) 23(11):1859–71. doi: 10.1093/neuonc/noab102 PMC8563320 33908609

[41] Dong Y Wang Q Sun J Liu H Wang H . Long non-coding RNA TPTEP1 exerts inhibitory effects on hepatocellular carcinoma by impairing microRNA-454-3p-mediated DLG5 downregulation. Dig Liver Dis (2022) 54(2):268–79. doi: 10.1016/j.dld.2021.04.014 34238665

[42] Zhuang X Veltri DP Long EO . Genome-wide CRISPR screen reveals cancer cell resistance to NK cells induced by NK-derived IFN-gamma. Front Immunol (2019) 10:2879. doi: 10.3389/fimmu.2019.02879 31921143 PMC6917608

[43] Liu DK Wang J Guo Y Sun ZX Wang GH . Identification of differentially expressed genes and fusion genes associated with malignant progression of spinal cord gliomas by transcriptome analysis. Sci Rep (2019) 9(1):13583. doi: 10.1038/s41598-019-50072-9 31537867 PMC6753211

[44] Wan S Lei Y Li M Wu B . A prognostic model for hepatocellular carcinoma patients based on signature ferroptosis-related genes. Hepatol Int (2022) 16(1):112–24. doi: 10.1007/s12072-021-10248-w 34449009

[45] Yang CB Feng HX Dai CL . Development and validation of an immune-related prognosis signature associated with hypoxia and ferroptosis in hepatocellular carcinoma. Cancer Med (2022) 00:1–13. doi: 10.1002/cam4.4556 PMC9160815 35092175

[46] Chen T Dai X Dai J Ding C Zhang Z Lin Z . AFP promotes HCC progression by suppressing the HuR-mediated Fas/FADD apoptotic pathway. Cell Death Dis (2020) 11(10):822. doi: 10.1038/s41419-020-03030-7 33009373 PMC7532541

