# Supplementary figures and images for: Analysis of cuproptosis in hepatocellular carcinoma using multi-omics reveals a comprehensive HCC landscape and the immune patterns of cuproptosis
Source: Front Oncol. 2022 Nov 2;12:1009036. doi: 10.3389/fonc.2022.1009036 (PMC9666696; doi:10.3389/fonc.2022.1009036)

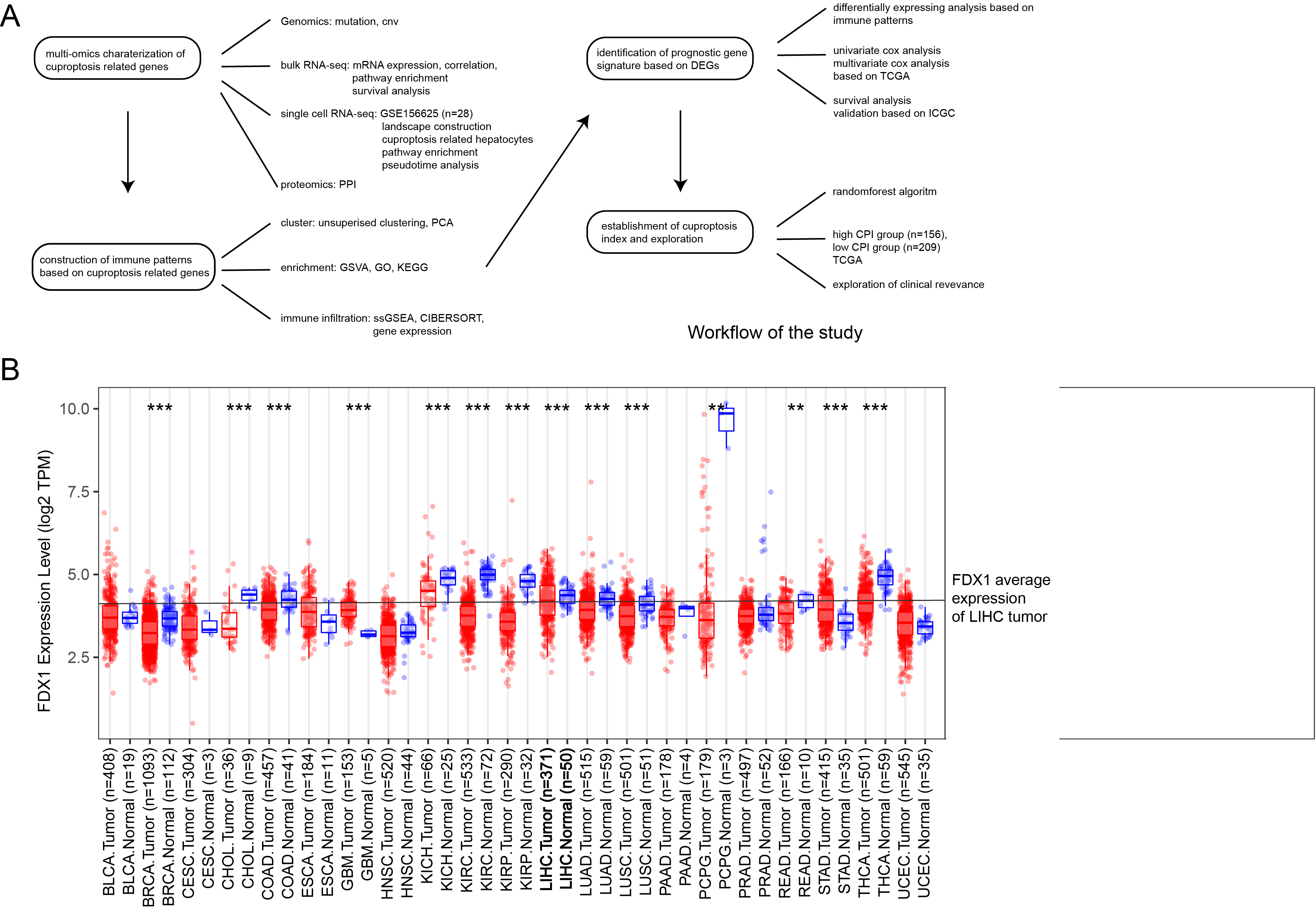

Supplement: Supplementary Figure 1 — Workflow of the study. (A) Route diagram showing the main flow of the study, including multi-omics characterizations of cuproptosis-related genes, construction of immune patterns based on cuproptosis-related genes, identification of prognostic gene signatures as based on DEGs and establishment and evaluation of the cuproptosis index. (B) Expression patterns of FDX1 in pan-cancer. (* p < 0.05; ** p < 0.01; *** p < 0.001). [file Image_1.tif]

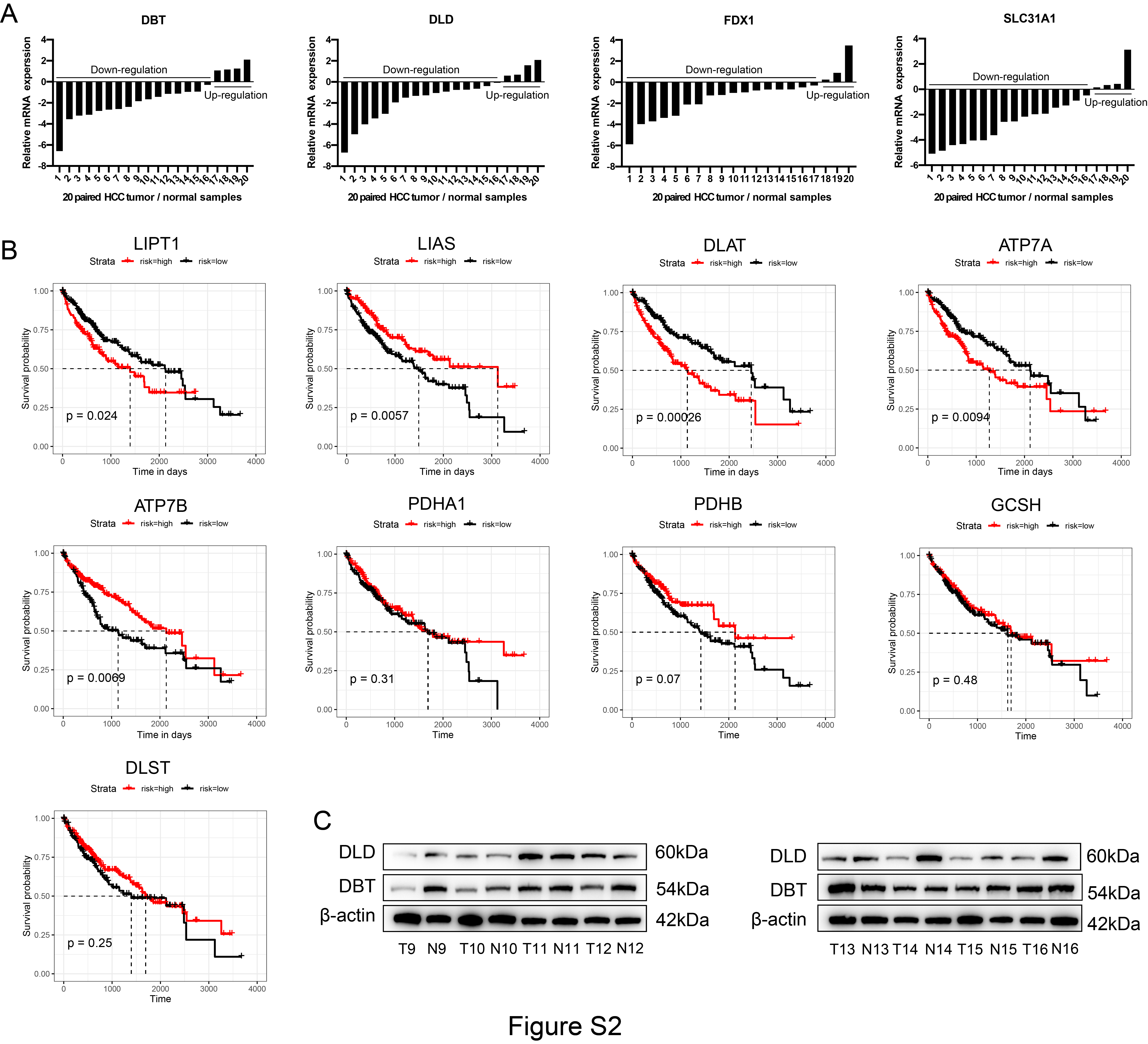

Supplement: Supplementary Figure 2 — QRT-PCR and survival analysis of cuproptosis-related genes in HCC. (A) qRT-PCR showed that compared with the adjacent normal tissues, FDX1, DBT, DLD and SLC31A1 were lowly expressed in tumor tissues. (B) Results of the KM analysis showing expressions of each cuproptosis-related gene that influenced the survival of TCGA. Red line represents the high-risk group and dark line the low-risk group. (C) the expression of DLD, DBT by Western blot in other 8 paired tissue samples, with differences between HCC and normal tissues. [file Image_2.tif]

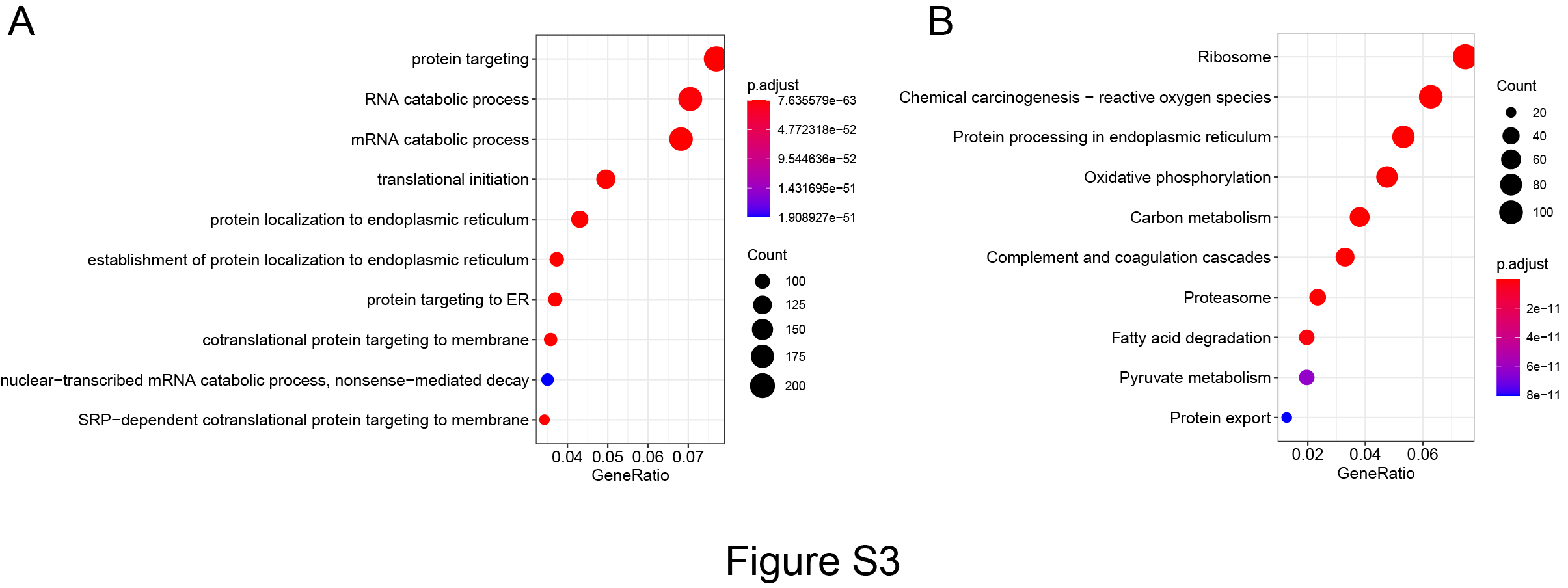

Supplement: Supplementary Figure 3 — Functional enrichment of cuproptosis-related hepatocytes. (A) Dot plots showing the top 10 GO terms enriched by DEGs in cuproptosis-related hepatocytes. (B) Dot plots showing the top 10 KEGG pathways enriched by DEGs in cuproptosis-related hepatocytes. [file Image_3.tif]

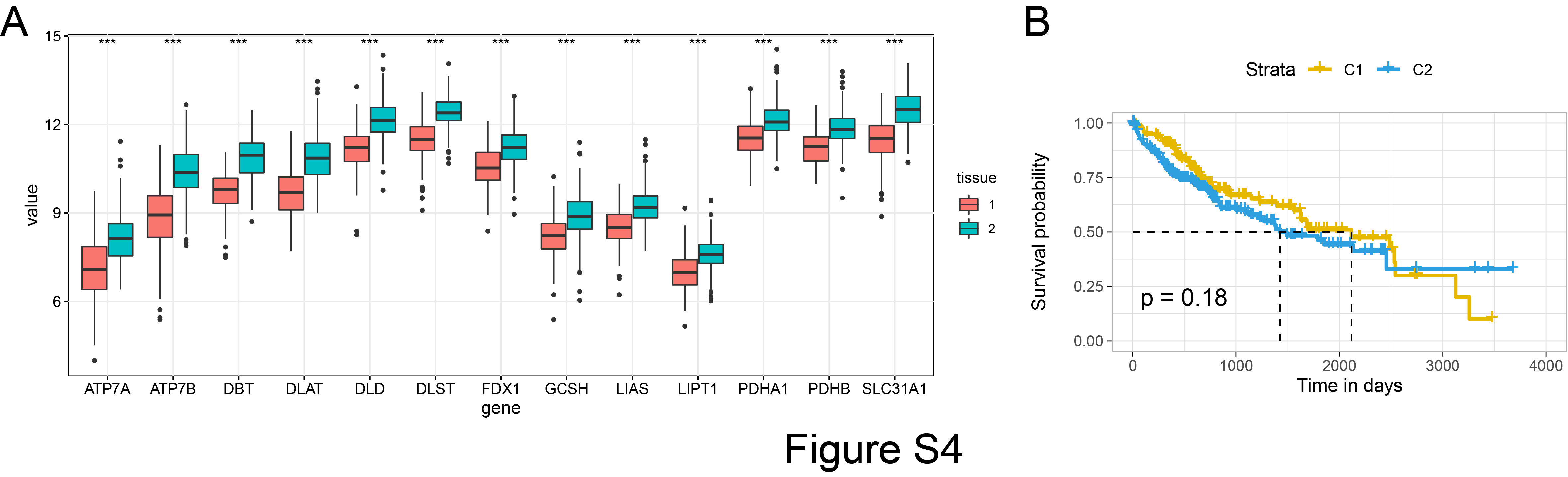

Supplement: Supplementary Figure 4 — Gene expression and survival analysis of cuproptosis patterns. (A) Boxplots showing the gene expression of 13 cuproptosis-related genes between the two cuproptosis patterns. (* p < 0.05; ** p < 0.01; *** p < 0.001). (B) Results of KM analysis showing survival probabilities for patients in cuproptosis-C1 and cuproptosis-C2 groups. Yellow line represents the Cuproptosis-C1 group and blue line the Cuproptosis-C2 group. [file Image_4.tif]

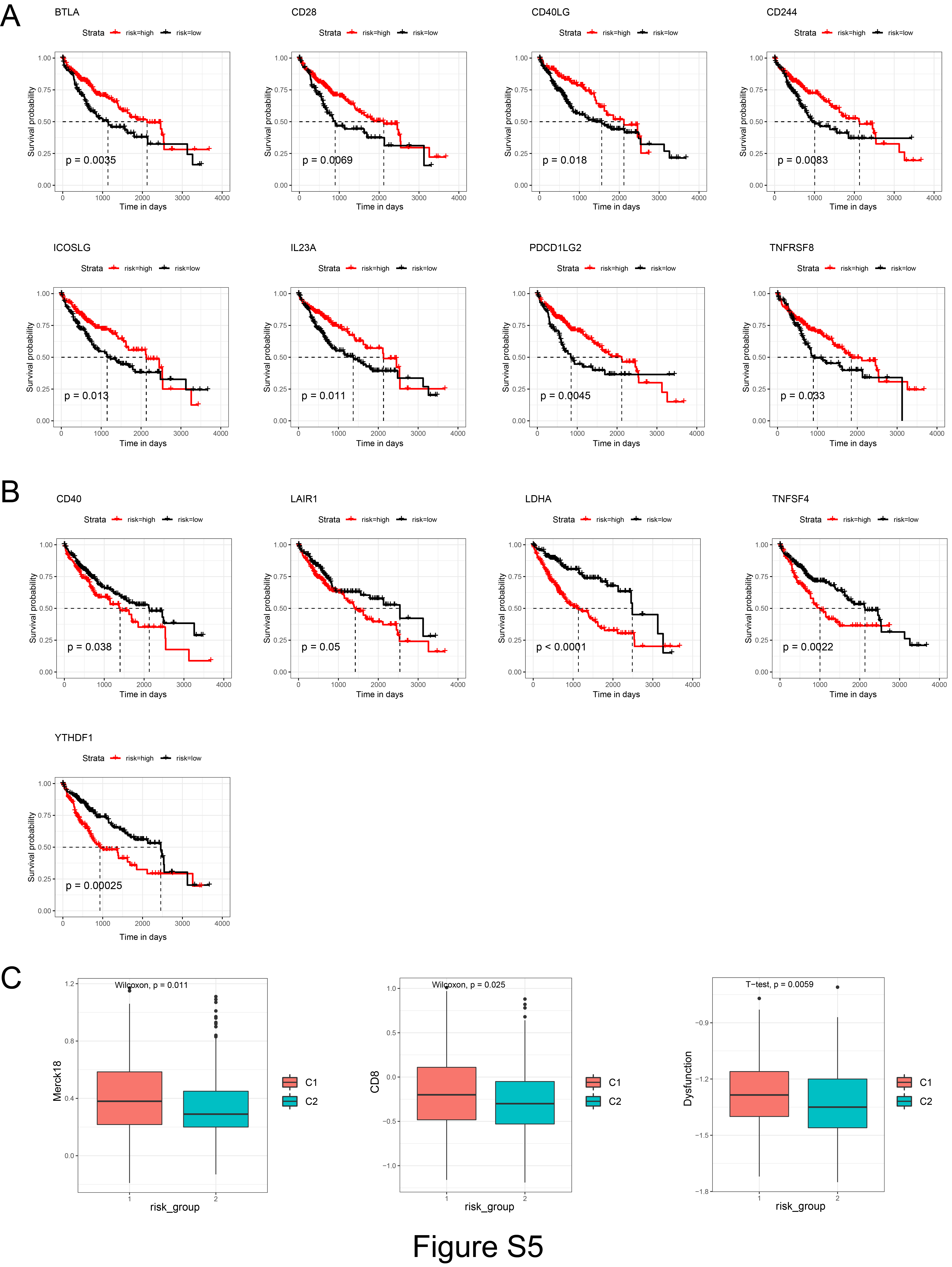

Supplement: Supplementary Figure 5 — Survival analysis of immune checkpoint genes. (A) Results of KM analysis showing high expressions of genes that were associated with a better prognosis: BTLA, CD28, CD40LG, CD244, ICOSLG, IL23A, PDCD1LG2 and TNFRSF8. Red line represents the high-risk group and dark line the low-risk group. (B) Results of KM analysis showing high expressions of genes associated with poor outcomes: CD40, LAIR1, LDHA, TNFSF4, YTHDF1. Red line represents the high-risk group and dark line the low-risk group. (C) Boxplots showing Merck18, CD8 and Dysfunction scores between two patterns. [file Image_5.tif]

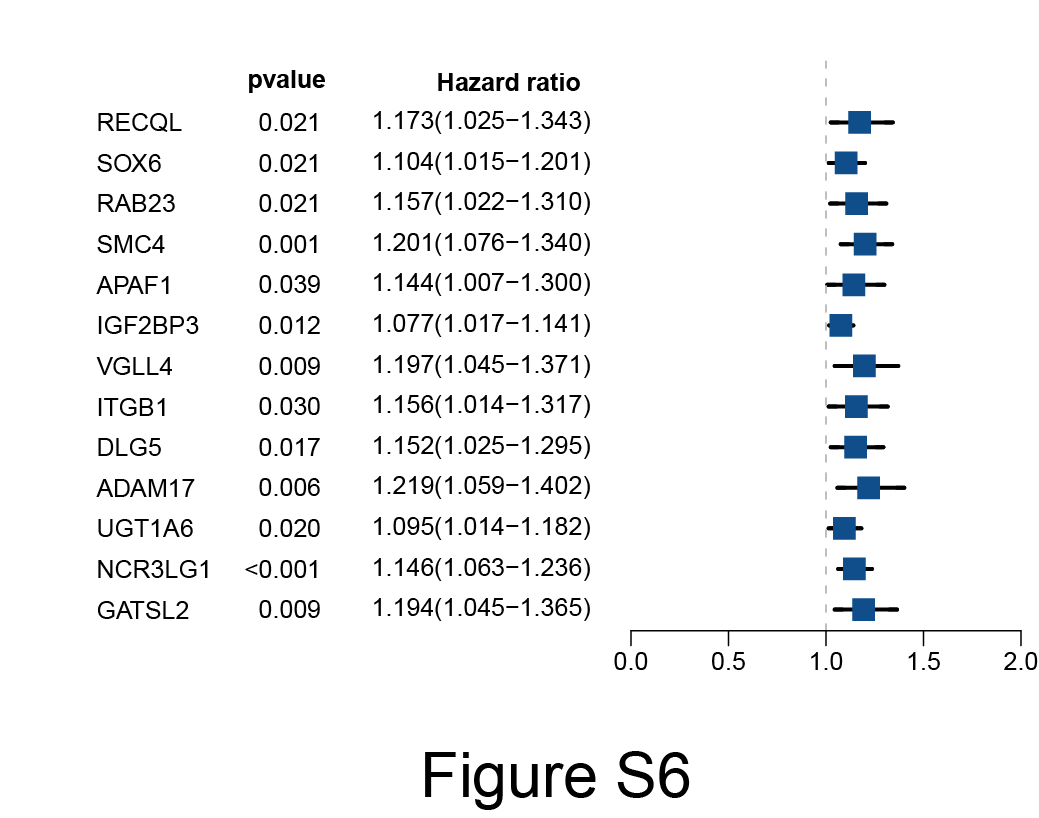

Supplement: Supplementary Figure 6 — Univariate cox analysis of DEGs by patterns. Forest plot showing results of univariate Cox regression of the 13 genes. The 95% confidence interval for each group was indicated by the length of the horizontal line. Hazard ratio (HR) of all patients was indicated by the vertical dotted line. [file Image_6.tif]

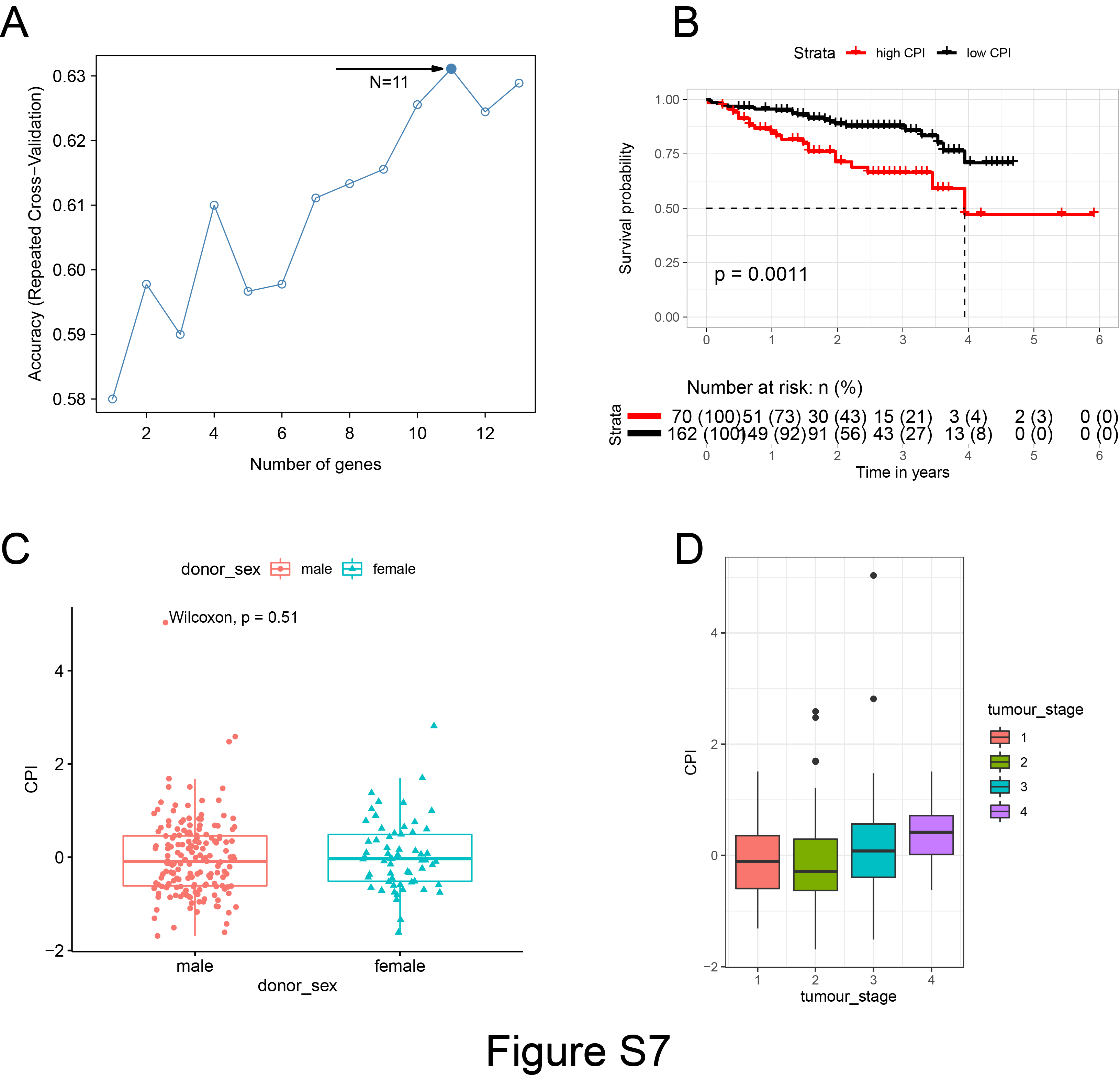

Supplement: Supplementary Figure 7 — Validation and evaluation of the cuproptosis index (CPI). (A) Line chart showing 11 genes exhibiting top accuracy for CPI using cross validation. (B) Results of KM analysis showing that CPI scores significantly influenced the survival of patients in ICGC. Red line represents the high-risk group and dark line the low-risk group. (C) CPI scores failed to indicate a statistically significant difference between genders. (D) Advanced tumor stage was related with high CPI scores. [file Image_7.tif]
